# Determinants of the Maternal 25-Hydroxyvitamin D Response to Vitamin D Supplementation During Pregnancy

**DOI:** 10.1210/jc.2016-2869

**Published:** 2016-10-28

**Authors:** Rebecca J. Moon, Nicholas C. Harvey, Cyrus Cooper, Stefania D'Angelo, Sarah R. Crozier, Hazel M. Inskip, Inez Schoenmakers, Ann Prentice, Nigel K. Arden, Nicholas J. Bishop, Andrew Carr, Elaine M. Dennison, Richard Eastell, Robert Fraser, Saurabh V. Gandhi, Keith M. Godfrey, Stephen Kennedy, M. Zulf Mughal, Aris T. Papageorghiou, David M. Reid, Sian M. Robinson, M. Kassim Javaid

**Affiliations:** Medical Research Council (MRC) Lifecourse Epidemiology Unit (University of Southampton) (R.J.M., N.C.H., C.C., S.D., S.R.C., H.M.I., E.M.D., K.M.G., S.M.R.), Southampton General Hospital; Paediatric Endocrinology (R.J.M.), University Hospital Southampton NHS Foundation Trust; and National Institute for Health Research (NIHR) Southampton Nutrition Biomedical Research Centre (N.C.H., C.C., K.M.G.), University of Southampton and University Hospital Southampton NHS Foundation Trust, Southampton SO16 6YD, United Kingdom; NIHR Musculoskeletal Biomedical Research Unit (C.C., N.K.A., A.C., M.K.J.), University of Oxford, Oxford OX3 7LD, United Kingdom; MRC Human Nutrition Research (I.S., A.P.), Elsie Widdowson Laboratory, Cambridge, United Kingdom CB1 9NL; Academic Unit of Child Health (N.J.B.), Sheffield Children's Hospital, University of Sheffield, Sheffield, United Kingdom S10 2TH; Academic Unit of Bone Metabolism (R.E.), University of Sheffield, Sheffield, United Kingdom S5 7AU; Sheffield Hospitals NHS Trust (University of Sheffield) (R.F., S.V.G.), Sheffield, United Kingdom S10 2SF; Nuffield Department of Obstetrics and Gynaecology (S.K., A.T.P.), John Radcliffe Hospital, University of Oxford, Oxford, United Kingdom OX3 9DU; Department of Paediatric Endocrinology (M.Z.M.), Royal Manchester Children's Hospitals, Manchester, United Kingdom M13 9WL; and School of Medicine and Dentistry (D.M.R.), Medical School, University of Aberdeen, Aberdeen, United Kingdom AB25 2ZD; Department of Medicine (I.S.), Norwich Medical School, Faculty of Medicine and Health Sciences, University of East Anglia, Norwich, United Kingdom NR4 7TJ

## Abstract

**Context:**

Current approaches to antenatal vitamin D supplementation do not account for interindividual differences in 25-hydroxyvitamin D (25(OH)D) response.

**Objective:**

We assessed which maternal and environmental characteristics were associated with 25(OH)D after supplementation with cholecalciferol.

**Design:**

Within-randomization-group analysis of participants in the Maternal Vitamin D Osteoporosis Study trial of vitamin D supplementation in pregnancy.

**Setting:**

Hospital antenatal clinics.

**Participants:**

A total of 829 pregnant women (422 placebo, 407 cholecalciferol). At 14 and 34 weeks of gestation, maternal anthropometry, health, and lifestyle were assessed and 25(OH)D measured. Compliance was determined using pill counts at 19 and 34 weeks.

**Interventions:**

1000 IU/d of cholecalciferol or matched placebo from 14 weeks of gestation until delivery.

**Main Outcome Measure:**

25(OH)D at 34 weeks, measured in a single batch (Diasorin Liaison).

**Results:**

25(OH)D at 34 weeks of gestation was higher in the women randomized to vitamin D (mean [SD], 67.7 [21.3] nmol/L) compared with placebo (43.1 [22.5] nmol/L; *P* < .001). In women randomized to cholecalciferol, higher pregnancy weight gain from 14 to 34 weeks of gestation (kg) (β = −0.81 [95% confidence interval −1.39, −0.22]), lower compliance with study medication (%) (β = −0.28 [−0.072, −0.48]), lower early pregnancy 25(OH)D (nmol/L) (β = 0.28 [0.16, 0.40]), and delivery in the winter vs the summer (β = −10.5 [−6.4, −14.6]) were independently associated with lower 25(OH)D at 34 weeks of gestation.

**Conclusions:**

Women who gained more weight during pregnancy had lower 25(OH)D in early pregnancy and delivered in winter achieved a lower 25(OH)D in late pregnancy when supplemented with 1000 IU/d cholecalciferol. Future studies should aim to determine appropriate doses to enable consistent repletion of 25(OH)D during pregnancy.

## Abstract (precis)

Within the treatment arm of the MAVIDOS RCT, poorer 25(OH)D response to 1000IU/d cholecalciferol was associated with lower initial 25(OH)D, greater pregnancy weight gain and delivery in winter.

Maternal vitamin D insufficiency during pregnancy is common (1, 2), and there is evidence that this might have detrimental effects on maternal health, fetal development (3, 4) and the long-term skeletal health of children (1, 3). Severe maternal vitamin D deficiency during pregnancy can result in symptomatic hypocalcaemia in the neonate (3). Associations have been reported between maternal 25-hydroxyvitamin D (25(OH)D) and obstetric complications, including preeclampsia, gestational diabetes, preterm birth, and offspring anthropometry, although the findings are inconsistent (3, 4) and require confirmation in randomized controlled trials. Nonetheless, the Institute of Medicine (IOM) has suggested that risk of vitamin D insufficiency, defined as a 25(OH)D less than 50 nmol/L, should be avoided during pregnancy (5), and this is supported by the recent Global Consensus on the Prevention of Rickets (6). Indeed many national guidelines recommend universal antenatal vitamin D supplementation to prevent vitamin D insufficiency (7–9).

Risk factors for vitamin D insufficiency are well described, and include ethnicity, extensive skin covering and liberal use of sun protection, overweight/obesity, low dietary vitamin D intake, and smoking (1, 10, 11), in addition to the seasonal variation that is observed at temperate latitudes (11, 12). Although vitamin D supplementation can improve maternal 25(OH)D status (10), little is known about how maternal characteristics might influence the 25(OH)D achieved after supplementation. In nonpregnant adults, baseline 25(OH)D concentration, body weight/adiposity and age are important determinants of the incremental rise in 25(OH)D after vitamin D supplementation (13, 14). During pregnancy, maternal hemodilution is accompanied by a number of physiological changes to both vitamin D metabolism (15) and maternal body composition (16); such adaptations might lead to differences in the determinants of response to vitamin D supplementation between pregnant and nonpregnant women. Clinically, understanding how individuals respond could lead to individualized antenatal counseling regarding vitamin D supplementation to ensure vitamin D repletion is achieved without increasing the risk of vitamin D toxicity. We therefore undertook this study to determine maternal characteristics associated with achieved 25(OH)D after antenatal vitamin D supplementation in the context of a double-blind, randomized, placebo-controlled trial.

## Materials and Methods

### The Maternal Vitamin D Osteoporosis Study (MAVIDOS)

The MAVIDOS study is a multicenter, double-blind, randomized, placebo-controlled trial of vitamin D supplementation in pregnancy. The primary outcome was neonatal bone mass. A detailed description of the study methods (17) and primary findings relating to offspring and maternal outcomes have been published previously (18). The study was approved by the Southampton and South West Hampshire Research Ethics Committee. MAVIDOS was registered prospectively (ISRCTN 82927713; EUDRACT 2007-001716-23); full approval from United Kingdom (UK) Medicines and Healthcare Products Regulatory Authority was granted, and written, informed consent was obtained from all participants.

Briefly, women attending one of 3 UK hospitals (University Hospital Southampton National Health Service (NHS) Foundation Trust, Southampton, UK [latitude 50.9° North]; Oxford University Hospitals NHS Foundation Trust, Oxford, UK [latitude 51.8° North]; Sheffield Hospitals NHS Trust [University of Sheffield], Sheffield, UK [latitude 53.4° North]) for early pregnancy ultrasound screening (11–14 wk of gestation) were invited to participate in the study. Inclusion criteria were: age over 18 years, singleton pregnancy, and gestation less than 17 weeks based on last menstrual period and ultrasound measurements. Women with known metabolic bone disease, renal stones, hyperparathyroidism or hypercalciuria, those taking medication known to interfere with fetal growth, fetal anomalies on ultrasonography, and women already using more than 400 IU/d vitamin D supplementation were excluded. A screening blood sample was obtained and analyzed on the local NHS platform (all 3 laboratories [Southampton, Oxford, and Sheffield] participate in Vitamin D External Quality Assessment Scheme vitamin D quality assurance system [http://www.deqas.org/]). Women with 25(OH)D between 25 and 100 nmol/L and serum calcium less than 2.75 mmol/L were eligible to enroll fully in the study.

Participants were randomized to either cholecalciferol 1000 IU/d or matched placebo (Merck KGaA/Sharp Clinical Services [previously DHP-Bilcare]), which was commenced before 17 weeks of gestation. The study medication was provided in a blister pack in a single box containing all medication for the whole pregnancy. All participants received standard antenatal care and could continue self-administration of dietary supplements containing up to 400 IU/d vitamin D.

### Maternal assessments during pregnancy

Before commencing the study medication, and again at 34 weeks of gestation, the women attended the research center for a detailed assessment of diet (including supplement use), lifestyle (smoking, physical activity participation, employment), and health (past medical history, current medication use) using interviewer-led questionnaires. Ethnicity was determined by participant self-report and subsequently categorized as White or non-White.

Anthropometric measurements included height, measured to the nearest 0.1 cm using a stadiometer, and weight, assessed to the nearest 0.1 kg using calibrated electronic scales. Four site (triceps, biceps, subscapular, and suprailiac) skinfold thicknesses were measured to the nearest 0.2 mm using a Harpenden skinfold caliper. Pregnancy weight gain was calculated as the difference between the weights at commencing study medication and 34 weeks of gestation.

### Compliance with study medication

Participants were asked to bring any remaining study medication to each assessment. The pills were counted and compliance calculated as number consumed/expected consumption based on number of days since medication was dispensed and expressed as a percentage. The 34 week visit was used for the calculation of compliance, and the count at 19 weeks was used if a 34-week count was not available.

### Assessment of 25(OH)D status

On the day that the study medication was dispensed and at 34 weeks of gestation, a nonfasted venous blood sample was obtained, and serum was stored at −80°C. 25(OH)D was assessed by RIA (Liaison RIA automated platform; Diasorin). All samples were analyzed in a single batch at the end of the study at Medical Research Council Human Nutrition Research. Details of assay performance and quality control through participation in Vitamin D External Quality Assessment Scheme, National Institute of Standards and Technology, and United Kingdom National External Quality Assessment Service are given elsewhere (19, 20).

### Statistical analysis

Women who had a measurement of 25(OH)D at both 14 and 34 weeks of gestation and delivered a live-born infant were included in the analysis (because pathology associated with fetal death might influence 25(OH)D concentrations). Maternal characteristics were compared between the women who did and did not complete the study using *t* tests, Mann-Whitney *U* tests, and χ^2^ tests for normally distributed, nonnormally distributed, and categorical variables, respectively. Linear regression was used to assess the association between maternal characteristics and 25(OH)D at 34 weeks of gestation for each treatment group separately. Multivariate linear regression was subsequently performed including all variables with a *P* < .2 from the linear regression. Additionally, maternal factors associated with achieving a vitamin D replete status (>50 nmol/L) were determined using Poisson regression with robust standard errors (21). The cut-point of 50 nmol/L as the definition for vitamin D replete status was chosen to reflect the IOM guidelines (5). Additionally, we considered a 25(OH)D more than 125 nmol/L as indicating risk of toxicity, as suggested by the IOM (5). In the primary trial analysis, we classified season of birth according to the UK Meteorological office recommendations (www.metoffice.gov.uk) with winter (December–February), spring (March–May), summer (June–August), and autumn (September–November). Because 25(OH)D concentrations are nonlinearly associated with season, to facilitate ready comparison, we collapsed this classification into 2 groups with a notional “winter” (the months in which 25(OH)D concentrations tended to be lowest, December-May) and a “summer” (the months in which 25(OH)D concentrations tended to be highest, June-November). Finally, in sensitivity analysis, we excluded women who reported having taken any additional vitamin D-containing supplements within 90 days of the late pregnancy blood sampling. All analyses were performed in Stata v14 (Statacorp). *P* < .05 was considered statistically significant.

## Results

**Figure 1.**
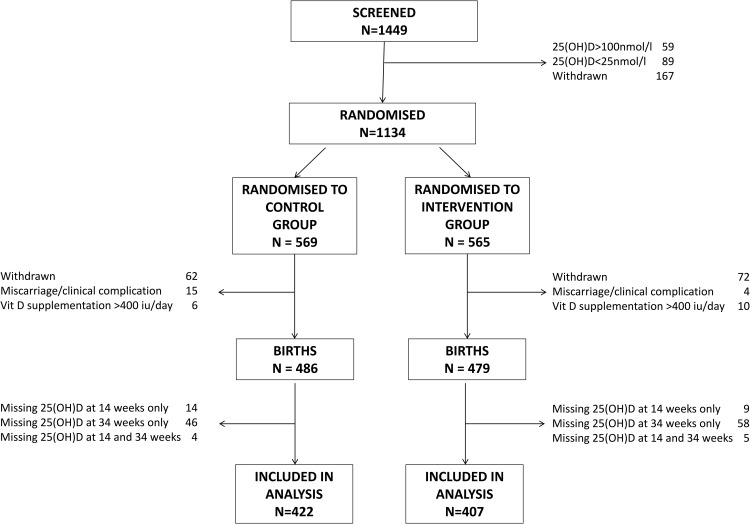
Consort diagram.

A total of 829 women, who delivered a live born infant and had measurements of 25(OH)D at both 14 and 34 weeks of gestation, were included in the analysis (Figure 1). Women with missing 25(OH)D measurements at 34 weeks, who delivered a live born infant (n = 136) were of similar age, parity, height, ethnicity, educational achievement, early pregnancy body mass index (BMI), and smoking status to those included in this analysis (*P* > .05 for all). There were no significant differences in baseline characteristics between women randomized to placebo and vitamin D supplementation (Table 1). Compliance with study medication was high in both treatment groups (placebo median 95% [interquartile range (IQR) 88%–98%], cholecalciferol median 96% [IQR 89%–99%], *P* = .11).

**Table 1.** Maternal Characteristics at Baseline According to Randomization Group

	Placebo	1000-IU/d Cholecalciferol
n	422	407
Gestation (wk), mean (SD)	15.9 (1.5)	15.9 (1.5)
Maternal age (y), mean (SD)	30.7 (5.4)	30.7 (5.0)
Nulliparous (%)	44.8	42.7
Current smoker (%)	7.7	7.7
BMI (kg/m^2^), median (IQR)	25.4 (22.7–29.7)	24.6 (22.2–28.6)
Height (cm), mean (SD)	165.6 (6.6)	165.5 (6.3)
White ethnicity (%)	94.8	95.6
25(OH)D (nmol/L), median (IQR)	44.4 (33.2–57.0)	45.7 (34.3–57.8)

### Maternal 25(OH)D status at 34 weeks of gestation by randomization group

Maternal 25(OH)D at 34 weeks of gestation was greater in the women randomized to cholecalciferol (mean 67.7 nmol/L [SD 21.3 nmol/L]) compared with the placebo group (mean 43.1 nmol/L [SD 22.5 nmol/L], *P* < .0001); 83.3% of women randomized to cholecalciferol achieved vitamin D replete status at 34 weeks of gestation (>50 nmol/L) compared with 35.6% in the placebo group (*P* < .001). Of the women who were not vitamin D replete at baseline (n = 509), 78.8% in the cholecalciferol group were replete at 34 weeks of gestation, compared with only 28.3% of the placebo group (*P* < .001). Similarly, only 48.4% of women who were vitamin D replete at baseline and received placebo remained vitamin D replete at 34 weeks of gestation, compared with 89.8% in the cholecalciferol group (*P* < .001). In both treatment groups, the proportion of women who were vitamin D replete at 34 weeks of gestation was lower in those who delivered in winter (Table 2). No participant reported symptoms suggestive of vitamin D toxicity. Two participants (0.5%) randomized to placebo and 1 to cholecalciferol (0.3%) (*P* difference = .58) had a 25(OH)D more than or equal to 125 nmol/L at 34 weeks of gestation, with the maximum value being 139 nmol/L.

**Table 2.** Percentage of Women Achieving Vitamin D Replete Status (>50 nmol/L) According to Randomization Group and Season of Delivery

Season of Delivery	Placebo	1000 IU/d Cholecalciferol	*P* Comparing Randomization Groups
Winter (December–May)	13.9	75.0	<.001
Summer (June–November)	54.2	90.1	<.001
*P* comparing seasons	<.001	<.001	

### Determinants of maternal 25(OH)D at 34 weeks of gestation

In univariate analysis, maternal age, baseline 25(OH)D, season of delivery and compliance with study medication were significantly associated with 34 week 25(OH)D in both the placebo and vitamin D supplementation groups (Table 3). Additionally, women who reported smoking in late pregnancy had significantly lower 25(OH)D in the placebo group, but this association was not observed among women randomized to cholecalciferol. Conversely, markers of maternal weight and adiposity were significantly inversely associated with maternal 25(OH)D in the cholecalciferol group but not the women randomized to placebo.

**Table 3.** 25(OH)D Status at 34 Weeks of Gestation According to Maternal Characteristics in Women Randomized to Placebo or Vitamin D Supplementation From 14 Weeks of Gestation

	Placebo		1000-IU/d Cholecalciferol	
	β (95% CI)	*P*	β (95% CI)	*P*
Maternal age (y)	**0.67 (0.28, 1.07)**	**.001**	**0.70 (0.28, 1.11)**	**.001**
Parity (yes vs no)	−1.25 (−5.69, 3.20)	.581	−0.29 (−4.60, 4.03)	.896
Smoking at 34 weeks of gestation (yes vs no)	**−13.45 (−22.12, −4.78)**	**.002**	−1.49 (−9.50, 6.52)	.715
Ethnicity (other vs White)	−8.69 (−18.59, 1.21)	.085	1.99 (−8.50, 12.48)	.709
Height (cm)	0.15 (−0.19, 0.48)	.389	−0.072 (−0.41, 0.27)	.675
BMI at 14 weeks of gestation (kg/m^2^)	−0.24 (−0.69, 0.20)	.284	**−0.47 (−0.90, −0.048)**	**.029**
Weight at 34 weeks of gestation (kg)	−0.056 (−0.22, 0.11)	.492	**−0.23 (−0.38, −0.085)**	**.002**
Weight gain early to late pregnancy (kg)	−0.23 (−0.85, 0.40)	.473	**−0.65 (−1.26, −0.039)**	**.037**
Triceps SFT at 34 weeks of gestation (mm)	−0.059 (−0.38, 0.26)	.718	**−0.42 (−0.74, −0.10)**	**.010**
Moderate/strenuous exercise in late pregnancy (h/wk)	1.36 (−1.75, 4.47)	.389	−0.76 (−3.63, 2.10)	.600
25(OH)D at 14 weeks of gestation (nmol/L)	**0.52 (0.40, 0.64)**	**<.001**	**0.21 (0.089, 0.33)**	**.001**
Season of delivery (summer vs winter)	**22.77 (19.05, 26.50)**	**<.001**	**10.09 (6.039, 14.15)**	**<.001**
Compliance (%)	**0.23 (0.044, 0.42)**	**.016**	**0.39 (0.19, 0.59)**	**<.001**

Shown as nmol/L change in 25(OH)D per unit predictor. Bold typeface highlights the findings that are statistically significant. SFT, skinfold thickness.

**Figure 2.**
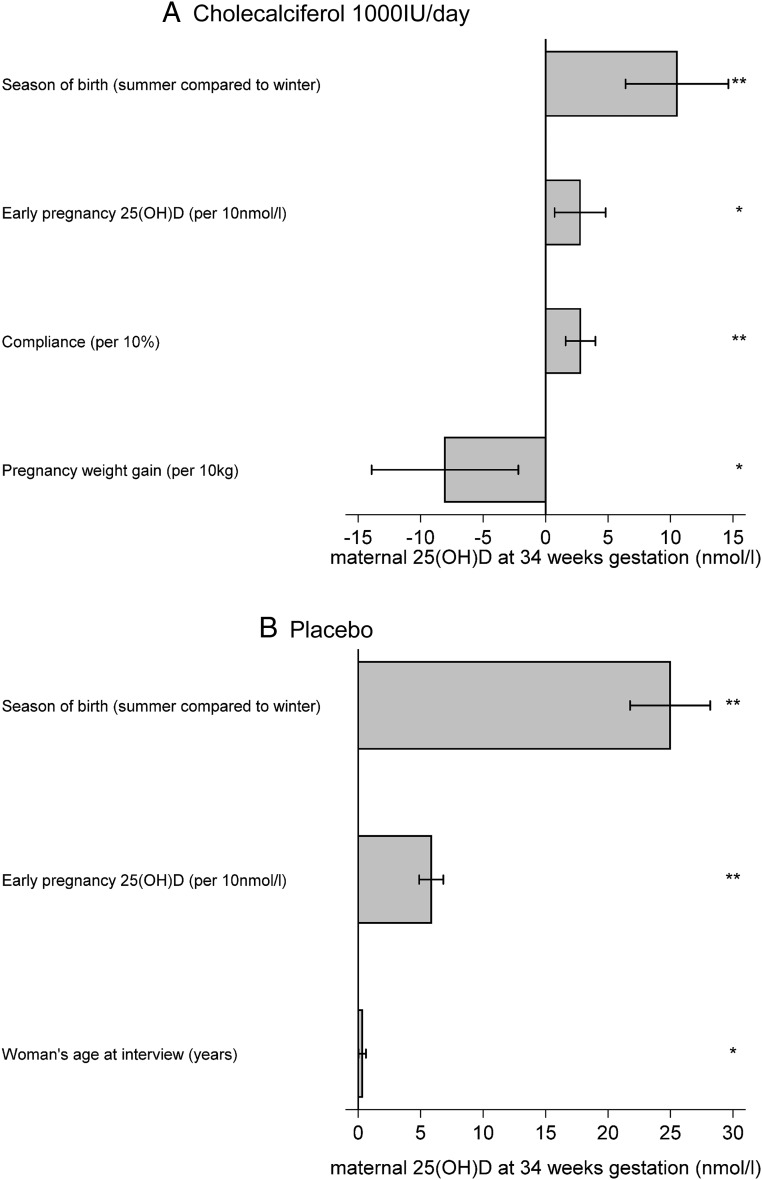
Independent determinants of maternal 25(OH)D at 34 weeks of gestation (A) after supplementation with 1000-IU cholecalciferol per day from 14 weeks of gestation until delivery and (B) receiving placebo from 14 weeks of gestation until delivery. Shown as change in 25(OH)D per unit predictor. *, *P* < .05; **, *P* < .01. A, Cholecalciferol 1000 IU/d. B, Placebo.

In multiple linear regression analysis, maternal factors significantly associated with greater 25(OH)D at 34 weeks of gestation in the vitamin D supplementation group were lower pregnancy weight gain (kg) (β = −0.81; 95% confidence interval [CI] −1.39, −0.22; *P* = .007), higher compliance (%) (β = 0.28; 95%CI 0.072, 0.48; *P* = .008), higher early pregnancy 25(OH)D (nmol/L) (β = 0.28; 95%CI 0.16, 0.40; *P* < .001), and summer delivery (summer vs winter) (β = 10.51; 95%CI 6.40, 14.63; *P* < .001) (Figure 2A). In the placebo group (Figure 2B), higher early pregnancy 25(OH)D (nmol/L) (β = 0.59; 95%CI 0.49, 0.68; *P* < .001), summer delivery (summer vs winter) (β = 24.97; 95%CI 21.77, 28.17; *P* < .001), and greater maternal age (y) (β = 0.32; 95%CI 0.022, 0.62; *P* = .04) remained significantly associated with greater 25(OH)D at 34 weeks of gestation. When achievement of vitamin D replete status at 34 weeks of gestation was considered instead of absolute 25(OH)D concentration, in multivariate analyses, delivery in summer (relative risk [RR] = 1.20; 95%CI 1.09, 1.33; *P* < .001), White ethnicity (RR = 1.27; 95%CI 1.17, 1.37; *P* < .001), greater compliance with medication (%) (RR = 1.01; 95%CI 1.00, 1.02; *P* = .03), and greater early pregnancy 25(OH)D concentration (nmol/L) (RR = 1.003; 95%CI 1.001, 1.006; *P* = .007) were significantly associated with achieving 25(OH)D >50 nmol/L in the women randomized to cholecalciferol.

### Interaction between baseline 25(OH)D and randomization group

When comparing achieved 25(OH)D at 34 weeks of gestation between placebo and cholecalciferol groups, it was apparent that there was a statistically significant interaction between baseline 25(OH)D and randomization group (*P* < .001). Thus, there was a smaller difference in 25(OH)D concentrations at 34 weeks of gestation between the placebo and treatment arms with increasing 25(OH)D at 14 weeks of gestation.

### Sensitivity analyses

As participants were permitted to continue taking daily vitamin D supplements containing up to 400 IU, in the sensitivity analysis we excluded 229 women (n = 117 randomized to cholecalciferol) who reported taking other vitamin D containing dietary supplements at the late pregnancy interview. Similarly, 81.0% of women randomized to cholecalciferol were vitamin D replete at 34 weeks of gestation, compared with 29.4% of women randomized to placebo (*P* < .001). The maternal characteristics associated with 25(OH)D at 34 weeks of gestation and achieving vitamin D replete status were similar to those observed in the whole cohort.

## Discussion

We have assessed anthropometric and demographic factors associated with the response to antenatal supplementation with 1000 IU/d cholecalciferol. This dose achieved vitamin D repletion in over 80% of women, without leading to 25(OH)D levels potentially associated with vitamin D toxicity (at least within the included baseline of 25–100 nmol/L 25(OH)D). However, gaining less weight during pregnancy, having a higher 25(OH)D in early pregnancy, delivering in summer and having higher compliance with supplementation were independently associated with achieving a greater 25(OH)D concentration in late pregnancy among women randomized to vitamin D supplementation. Thus, those women who are at risk of vitamin D insufficiency in early pregnancy, gain greater weight, and deliver in winter might need supplementation with a higher dose of cholecalciferol to achieve similar 25(OH)D concentrations. However, when vitamin D replete status was considered as the outcome, only non-White maternal ethnicity and delivery in winter were significant predictors of vitamin D nonreplete status after supplementation.

To our knowledge, the factors which determine the response to vitamin D supplementation in pregnancy have not previously been assessed. However, our findings are consistent with those in nonpregnant adults (13, 14). It is well recognized that individuals who are overweight or obese are at higher risk of vitamin D insufficiency, and this is similarly observed in pregnancy (2, 22). Studies in nonpregnant adults have also shown that obese individuals achieve a lower 25(OH)D with the same dose of supplementation as nonobese individuals (14). Metaanalysis of vitamin D supplementation studies has suggested that over 50% of the variance in 25(OH)D increment in response to supplementation is explained by body weight (13). Although the relationship between body weight and 25(OH)D increment after supplementation could reflect sequestration in adipose tissue, we found that in multivariate analysis, prepregnancy BMI and late pregnancy triceps skinfold thickness (as a marker of adiposity), were not associated with 25(OH)D after supplementation, but that pregnancy weight gain was negatively associated. Similarly, we have previously demonstrated that greater gestational weight gain is associated with a decline in 25(OH)D status during pregnancy, independent of supplement use (12). Weight gain in pregnancy represents not only increased fat mass but also feto-placental tissues and hemodilution (16); our data, therefore, suggest that overall volume of dilution, and not just adiposity, may be important for response to vitamin D supplementation. However, importantly, when using a 25(OH)D more than 50 nmol/L as a cut-point for repletion, pregnancy weight gain was not an independent predictor of achieving vitamin D repletion.

Despite receiving 1000 IU cholecalciferol per day, 25% of mothers delivering in winter had a 25(OH)D less than 50 nmol/L. This is a higher nonrepletion rate than that reported in other recent pregnancy supplement studies (23–25). However, it is notable that there were marked differences in baseline 25(OH)D concentrations between these investigations, and we observed that initial 25(OH)D status was positively associated with both the likelihood of achieving vitamin D replete status and absolute 25(OH)D status at 34 weeks of gestation. Importantly, the difference between the 25(OH)D achieved at 34 weeks of gestation in women randomized to placebo compared with cholecalciferol decreased with increasing baseline 25(OH)D. This is consistent with previous studies in adults, which have shown that the incremental response to vitamin D supplementation is higher in vitamin D insufficient than replete subjects (13, 14) and that the increase in 25(OH)D relative to supplementation dose is negatively associated with dose of vitamin D supplement (26). This suggests that physiological processes such as saturation of the hepatic 25-hydroxylase involved in the conversion of cholecalciferol to 25(OH)D or conversion to 24- or 4-OH metabolites, together with renal catabolism, limit attainment of very high 25(OH)D concentrations (27). This mechanism might be important in preventing hypervitaminosis D. However, studies comparing the effectiveness of differing doses of vitamin D in pregnancy have shown that 4000 IU/d can achieve a higher 25(OH)D than 400 IU/d (10, 28), but whether these higher doses are of clinical benefit is yet to be demonstrated (4, 29) and at the general population level, lower doses would be compatible with keeping 25(OH)D below a concentration which might be concerning.

It is evident from our findings that 1000 IU/d cholecalciferol in pregnancy does not eliminate the seasonal variation in 25(OH)D status observed in pregnant women in the UK (11, 12). Similarly, non-White ethnicity was associated with a higher risk of not achieving vitamin D replete status in the supplemented women. Hollis et al similarly found that even with 4000 IU/d vitamin D during pregnancy, African-American women had lower 25(OH)D in late pregnancy than Caucasian or Hispanic women (10). Thus, future studies should aim to determine the dose required to achieve optimal 25(OH)D status among women of non-White ethnicity and among those who deliver in winter months. Maternal age was also positively associated in univariate analyses with 25(OH)D achieved at 34 weeks of gestation in women who received vitamin D supplementation. It has previously been shown in pregnant women that age is positively associated with 25(OH)D status (30, 31). Although lower uptake of supplementation in younger women (12) could partly explain this observation, our finding would additionally suggest that even in younger women who do use supplements, the achieved 25(OH)D is lower. Data from healthy and hospitalized adults have similarly shown that older individuals achieve a higher 25(OH)D after vitamin D supplementation (13, 32). As such, young pregnant women might particularly require advice on the need for, and compliance with, vitamin D supplementation.

Although our findings are novel, and may enable the development of individualized advice for antenatal vitamin D supplementation, there are a number of limitations which should be considered in the interpretation of this study. Firstly, we could not, as a result of stipulations made during the ethics approval process, include participants with 25(OH)D concentrations less than 25 nmol/L or more than 100 nmol/L. As baseline 25(OH)D was associated with the likelihood of achieving vitamin D replete status, it is likely that women with very low levels of 25(OH)D at baseline will require a higher supplementation dose to achieve vitamin D repletion. However, this needs to be confirmed in future studies. Secondly, only a small proportion of the women included in this study were of non-White ethnicity. This reflects the local populations and care should be taken in translating these findings to a more ethnically diverse population. Thirdly, we did not examine genetic determinants of the response to vitamin D supplementation. It has been demonstrated previously that the incremental rise in 25(OH)D after supplementation differs by single nucleotide polymorphisms in vitamin D binding protein (33, 34) and 25-hydroxylase genes (33). Although this genetic information can enable a more comprehensive understanding of the biochemical response to vitamin D supplementation, the current inability to undertake genotyping on a widespread population basis means this additional information would not allow for alterations to current clinical practice regarding vitamin D supplementation in pregnancy. Finally, during pregnancy, a number of physiological changes occur to vitamin D metabolism, including an increase in vitamin D binding protein and 1,25-dihydroxyvitamin D (15), indices that we were not able to include in our analysis.

In conclusion, we have demonstrated that women who gain more weight during pregnancy, have lower 25(OH)D in early pregnancy, or deliver in winter tend to achieve a lower 25(OH)D in late pregnancy when supplemented with 1000-IU/d cholecalciferol than do women with the converse attributes. Future studies should aim to determine appropriate doses to enable consistent repletion of 25(OH)D during pregnancy, and our findings support the notion that clinical approaches to vitamin D repletion may be informed by individual characteristics. As such, personalized vitamin D supplementation advice might become part of future antenatal care.
